# Prevalence and Risk Factors for Microalbuminuria in Children with Sickle Cell Disease at King Abdulaziz University Hospital: A Retrospective Cross-sectional Study

**DOI:** 10.7759/cureus.6638

**Published:** 2020-01-12

**Authors:** Yahya A Alzahrani, Malak A Algarni, Maryam M Alnashri, Hanan M AlSayyad, Khadijah M Aljahdali, Joud E Alead, Yara A Alhjrsy, Fatma Alzahrani, Osama Safdar

**Affiliations:** 1 Pediatrics, King Abdulaziz University Hospital, Jeddah, SAU; 2 Pediatrics, Family Medicine, Ibn Sina National College for Medical Studies, Jeddah, SAU; 3 Pediatrics, King Abdulaziz University, Jeddah, SAU

**Keywords:** sickle cell disease, microalbuminuria, retrospective study

## Abstract

Objectives: Previous studies have not addressed microalbuminuria in pediatric patients with sickle cell disease (SCD) in Jeddah, Saudi Arabia. This study aimed to determine the prevalence of microalbuminuria and to identify associated risk factors in children with SCD at King Abdulaziz University Hospital.

Results: Overall, 42.5% of the patients enrolled were Saudi Arabian and 51% were male. The mean age was 12.4 years, and the highest percentage (40%) was in the age group of 15-18 years. The prevalence of microalbuminuria was 9.6%, and hematuria was present in 8% of cases. The percentage of patients with hematuria was significantly higher in the microalbuminuria group (22.6%) than in the nonmicroalbuminuria group (6.5%; P = 0.007). The percentage of patients with acute chest syndrome was also higher in the microalbuminuria group (26%) than in the nonmicroalbuminuria group (8%; P = 0.005). The percentage of patients with gallbladder stones was higher in the microalbuminuria group (13%) than in the nonmicroalbuminuria group (2.4%; P = 0.014). However, the mean number of blood transfusions was higher in the nonmicroalbuminuria group than in the microalbuminuria group (P = 0.002). Sickle cell nephropathy manifests as microalbuminuria, begins at an early age, occurs in all types of SCD, and is associated with disease severity.

## Introduction

Sickle cell disease (SCD) is one of the most important autosomal recessive diseases. In the Kingdom of Saudi Arabia (KSA), the prevalence of the sickle cell trait ranges from 2% to 27%, and as many as 2.6% of affected individuals develop SCD. SCD is characterized by vaso-occlusive events, hemolytic crises, and organ damage [1].

Renal impairment is a chronic complication of SCD and a major factor associated with mortality [2]. This association with mortality is stronger than that observed with an episode of acute stroke, a febrile episode with positive blood culture, acute chest syndrome, or severe acute anemia [3].

In SCD, microalbuminuria is one of the most common clinical manifestations of sickle cell nephropathy (SCN) [4-5], which appears to be associated with a more rapid deterioration in renal function [6]. The reported incidence of microalbuminuria in children with SCD ranges from 18.4% to 46% [7-9]. The identification of microalbuminuria in a patient with SCD is a predictor of end-organ disease, including renal damage [10-11]. Children with SCD experience hyperfiltration and hyperperfusion, which are associated with renal damage [12-13]. Therefore, the early detection of microalbuminuria may represent an important early sign of renal disease [14].

A prolonged period of microalbuminuria precedes persistent proteinuria, which is followed by renal failure in SCD patients [15]. Therefore, the identification of risk factors for microalbuminuria may allow earlier intervention to prevent renal complications [7]. The KSA faces a high burden of SCD, with a prevalence rate of 2.6% in newborns in a population of >24 million, and children with SCD are prone to developing microalbuminuria and chronic renal failure with advancing age. Nevertheless, previous research in the KSA has not addressed this problem. Therefore, this study aimed to determine the prevalence of microalbuminuria in children with SCD and to identify the risk factors associated with microalbuminuria in children with SCD at King Abdulaziz University Hospital (KAUH).

## Materials and methods

The study was approved by the institutional review board of the KAUH (reference number: 186-19). This cross-sectional prevalence study retrospectively reviewed medical records of children aged 2-18 years who were diagnosed with SCD and visited the KAUH Pediatric Sickle Cell Clinic.

The following data were obtained from recent outpatient follow-up visits: sex, age, nationality, weight, height, ABO blood group type, sickle cell genotype, blood transfusion (BT) status, and number of transfusions. Additionally, the frequencies of vaso-occlusive events and SCD complications were collected.

From the urinalysis results, microalbuminuria was defined as a protein level of >1+. Hematuria was defined as a RBC count >5.

Statistical analysis

Descriptive statistics was used to assess the study participants’ demographic characteristics. Means ± standard deviations (SDs) and median values were used to describe continuous variables. Frequencies with proportions are used to report categorical variables. Numerical variables were compared between groups using the independent t-test, whereas categorical variables were compared using the chi-square and Fisher’s exact tests. Statistical significance was set at a P value <0.05. All statistical analyses were performed using IBM SPSS statistics, version 23 (IBM, Armonk, NY, USA).

## Results

The prevalence of microalbuminuria and its associated factors were assessed in 322 pediatric patients with SCD. The patients' characteristics are presented in Table 1.

**Table 1:** Descriptive statistics of all patients enrolled in the study (N=322). SD, standard deviation

Variable		n	Percentage
Nationality			
Non-Saudi Arabian		185	57.5
Saudi Arabian		137	42.5
Gender			
Female		157	48.8
Male		165	51.2
Age group			
2 to 5 years		30	9.3
6 to 10 years		87	27.0
11 to 14 years		77	23.9
15 to 18 years		128	39.8
Sickle cell genotype			
Hemoglobin SB 0 (Beta-zero) thalassemia		4	1.2
Hemoglobin SB+ (beta) thalassemia		41	12.7
Hemoglobin SS disease (sickle cell disease)		233	72.4
Sickle cell trait (hemoglobin S disease)		44	13.7
Microalbuminuria			
No		291	90.4
Yes		31	9.6
Hematuria			
No		296	91.9
Yes		26	8.1
Blood type			
A RhD negative (A-)		3	0.9
A RhD positive (A+)		84	26.1
AB RhD positive (AB+)		15	4.7
B RhD negative (B-)		3	0.9
B RhD positive (B+)		32	9.9
O RhD negative (O-)		9	2.8
O RhD positive (O+)		176	54.7
Blood transfusions			
No		143	44.4
Yes		179	55.6
Frequency of:			
Pneumonia		30	9.3
Priapism		3	0.9
Avascular necrosis		3	0.9
Acute chest syndrome		31	9.6
Aplasia		2	0.6
Stroke		14	4.3
Acute coronary syndrome		10	3.1
Dactylitis		3	0.9
Spleen sequestration		24	7.5
Gallbladder stones		11	3.4
Osteomyelitis		23	7.1
	Mean	SD	Median
Age (years)	12.43	4.64	13.00
Number of blood transfusions	8.67	26.71	1.00

The characteristics of the nonmicroalbuminuria (291 patients) and microalbuminuria group (31 patients) and comparisons of different variables between the groups are shown in Table 2. The prevalence of hematuria differed significantly between the groups, and was higher in the microalbuminuria group than in the nonmicroalbuminuria group (P = 0.007). However, a significant difference was observed in the distribution of blood groups (P = 0.022). The percentage of acute chest syndrome was significantly higher in the microalbuminuria group than in the nonmicroalbuminuria group (P = 0.005). The percentage of gallbladder stones was significantly higher in the microalbuminuria group than in the nonmicroalbuminuria group (P = 0.014). The mean number of BTs was higher in the nonmicroalbuminuria group than in the microalbuminuria group (P = 0.002). No other variables differed significantly between the groups.

**Table 2:** Comparison of different variables between nonmicroalbuminuria and microalbuminuria patients. SD, standard deviation; *, significant P-value

	Without microalbuminuria (n=291)		With microalbuminuria (n=31)			P-value		
	n	%	n	%				
Nationality								
Non-Saudi Arabian	167	57.4	18	58.1		0.942		
Saudi Arabian	124	42.6	13	41.9				
Sex								
Female	144	49.5	13	41.9		0.424		
Male	147	50.5	18	58.1				
Age group								
2 to 5 years	27	9.3	3	9.7		0.432		
6 to 10 years	82	28.2	5	16.1				
11 to 14 years	70	24.1	7	22.6				
15 to 18 years	112	38.5	16	51.6				
Sickle cell genotype								
Hemoglobin SB 0 (beta-zero) thalassemia	4	1.4	0	0.0		0.928		
Hemoglobin SB+ (beta) thalassemia	37	12.7	4	12.9				
Hemoglobin SS disease (sickle cell disease)	210	72.2	23	74.2				
Sickle cell trait (Hemoglobin S disease)	40	13.7	4	12.9				
Hematuria								
No	272	93.5	24	77.4		0.007*		
Yes	19	6.5	7	22.6				
Blood type								
A RhD negative (A-)	2	0.7	1	3.2		0.022*		
A RhD positive (A+)	75	25.8	9	29.0				
AB RhD positive (AB+)	10	3.4	5	16.1				
B RhD negative (B-)	3	1.0	0	0.0				
B RhD positive (B+)	31	10.7	1	3.2				
O RhD negative (O-)	9	3.1	0	0.0				
O RhD positive (O+)	161	55.3	15	48.4				
Blood transfusions								
No	131	45.0	12	38.7		0.502		
Yes	160	55.0	19	61.3				
Frequency of:								
Pneumonia	28	9.6	2	6.5		0.752		
Priapism	3	1.0	0	0.0		1		
Avascular necrosis	3	1.0	0	0.0		1		
Acute chest syndrome	23	7.9	8	25.8		0.005*		
Aplasia	2	0.7	0	0.0		1		
Stroke	14	4.8	0	0.0		0.377		
Acute coronary syndrome	10	3.4	0	0.0		0.607		
Dactylitis	3	1.0	0	0.0		1		
Spleen sequestration	22	7.6	2	6.5		1		
Gallbladder stones	7	2.4	4	12.9		0.014*		
Osteomyelitis	20	6.9	3	9.7		0.474		
	Without microalbuminuria			With microalbuminuria				P-value
	Mean	SD	Median	Mean	SD		Median	
Age	12.29	4.62	12.00	13.74	4.68		15.00	0.098
Number of transfusions	9.26	27.97	1.00	3.13	5.60		1.00	0.002*

## Discussion

Secondary renal failure affects 5%-20% of adult patients with SCD, and the progression of renal disorder begins in childhood [16]. Microalbuminuria is one of the earliest manifestations of SCN. Hence, many studies have aimed to determine the prevalence of microalbuminuria among SCD patients as an indicator of the severity of the condition [17-18]. In this study, we determined the prevalence of microalbuminuria of 9.6% among pediatric patients with a mean age of 12.4 years. This condition emerged at a very young age (2 years) and increased continuously to the highest percentage among young adults (15-18 years), who exhibited a prevalence of 51.6%. The mean age and average prevalence of microalbuminuria among older patients in our study were consistent with the prevalence rates of 46% as reported by Dharnidharka et al. [17] and of 39%-43% in adults with SCD as reported by McBurney et al. [7]. However, the overall prevalence of microalbuminuria among all patients (9.6%) was lower than the average prevalence reported by those previous studies. Alkhunaizi et al. [19] determined that the prevalence of microalbuminuria among adult Saudi Arabian patients (>18 years) was 25%, very similar to our findings in the same age group.

Dharnidharka et al. [17] and McBurney et al. [7] reported that no microalbuminuria was detected in children aged <7 years. Conversely, 9.7% of microalbuminuria patients in our study were aged 2-5 years. Our findings were supported by those of Aloni et al. [20] who confirmed the presence of microalbuminuria in patients aged <7 years. This early deterioration of glomerular function could be explained by the presence of certain factors, including a genetic predisposition, fetal hemoglobin (HbF) level, environmental factors, efficacy of medical care, and lifestyle factors associated with developing countries [21]. However, the small sample size in our study may also reasonably explain these contradictory results. The studies by Dharnidharka et al. [17] and by McBurney et al. [7] enrolled 104 and 151 patients, respectively. Interestingly, when we compared the microalbuminuria and nonmicroalbuminuria groups, we observed no significant difference in terms of age (P = 0.432), suggesting that this was not a defining variable in either group. Nevertheless, age was a defining variable in the progression of microalbuminuria in the affected group.

Previous publications have reported a female predominance of microalbuminuria. Eke et al. reported a microalbuminuria prevalence of 9.7% among female patients and 6.1% among male patients [5], while Okpere et al. [22] reported results consistent with female predominance (45.3% vs. 20.4% of males). We did not observe a significant difference in sex between the microalbuminuria and nonmicroalbuminuria groups in our study, consistent with the findings of McBurney et al. [7] and Dharnidharka et al. [17]. Consequently, additional research evidence is needed to clarify these contrasting results.

Our findings demonstrated that microalbuminuria occurs in association with most hemoglobin genotypes. The highest percentage was observed with the Hb-ss genotype (74.2%) in the microalbuminuria group, similar to the results of a previous study conducted by Wigfall et al. [23]. No microalbuminuria was detected in the HB-Sβ0 (Beta-Zero) thalassemia sub-group. Most previous studies included few patients with Sβ-thalassemia, and only a few studies have published mixed results regarding this patient group. Becton et al. [18] reported that only one patient with Sβ-thalassemia had microalbuminuria.

We further examined the frequencies of several clinical complications that may be associated with microalbuminuria (Table 2). We compared the microalbuminuria and nonmicroalbuminuria groups to identify definitive variables that varied significantly between the groups. Interestingly, we found that most patients in the microalbuminuria group experienced acute chest syndrome, gallbladder stones, osteomyelitis, pneumonia, and spleen sequestration, whereas none reported priapism, avascular necrosis, aplasia, stroke, acute coronary syndrome (ACS), or dactylitis. These findings were consistent with those reported by Dharnidharka et al. [17] and McBurney et al. [7] who observed no significant correlation between microalbuminuria and stroke and those of McBurney et al. [7] and Becton et al. [18] who reported no significant correlation with ACS. Our observation of a significant association between acute chest syndrome and microalbuminuria (P = 0.005) was consistent with the findings reported by Alvarez et al [24]. By contrast, Bodas et al. [25] reported that the glomerular filtration rate was not correlated with episodes of either stroke or acute chest syndrome, suggesting that the etiologies of these complications may differ from the etiologies underlying the development of SCN. However, that study included only 48 patients, and the relatively small sample size likely influenced the significant correlation between the two conditions.

We further identified a significant correlation between microalbuminuria and the development of gallbladder stones (P = 0.014). Our findings were consistent with those of Alexander-Reindorf et al. [26] and Bond et al. [27] who reported significantly higher morbidity and more hospital admissions among SCD patients with gallbladder stones. Additionally, the mean age in our microalbuminuria group was 13.74 years, consistent with the findings of Martins et al. [28] who reported patients of ages 11 and 29 years, with a higher prevalence of cholelithiasis and gallbladder stones respectively.

In our study, the number of BTs was significantly and negatively associated with microalbuminuria, suggesting that BTs are a renoprotective process in the management of SCD. Alvarez et al. [24] reported similar results and suggested that the early initiation of transfusion could protect the kidney and hinder deterioration of the SCN. However, the side effects of transfusion, such as iron overload, must be considered before starting this process. By contrast, Aloni et al. [20] reported that BT is not a significant factor with respect to microalbuminuria.

Becton et al. [18] stated that 36% of SCD patients presented with hematuria. However, the authors reported no significant difference in the frequency of hematuria between the microalbuminuria and nonmicroalbuminuria groups. By contrast, we observed a statistically significant difference in the frequency of hematuria between patients with and without microalbuminuria (P = 0.007). Our results were consistent with the findings of Sesso et al. [29] who reported higher frequencies of hematuria in the Hb-SS and Hb-AS groups. The authors stated that hematuria is caused by the increased sickling of RBCs in the renal medulla, resulting in extravasation and ischemia. We further determined that most patients with SCD had type O RhD+ blood and that this variable varied significantly between the two groups (P = 0.022). This result was consistent with the findings of Alagwu et al. [30] who reported an O blood group frequency of 63% among Hb-ss patients. This finding could be explained by the fact that the O Rh+ blood group is the most prevalent group in humans.

## Conclusions

In conclusion, our findings highlight the importance of early investigations (e.g., urinalysis) for the assessment of microalbuminuria and hematuria, as well as the determination of the degree of SCN. The observation that the average number of BTs was significantly higher in the nonmicroalbuminuria group than in the microalbuminuria group could suggest a protective role of transfusion against the development of microalbuminuria. Nevertheless, further investigations are needed to confirm our results. We also reported significantly higher rates of acute chest syndrome and gallbladder stones in patients with microalbuminuria. These factors must be considered, and special care should be provided to affected patients. We recommend routine screening of SCD patients for microalbuminuria and hematuria.
